# Supplementary figures and images for: An updated phylogeny of the Alphaproteobacteria reveals that the parasitic Rickettsiales and Holosporales have independent origins
Source: eLife. 2019 Feb 25;8:e42535. doi: 10.7554/eLife.42535 (PMC6447387; doi:10.7554/eLife.42535)

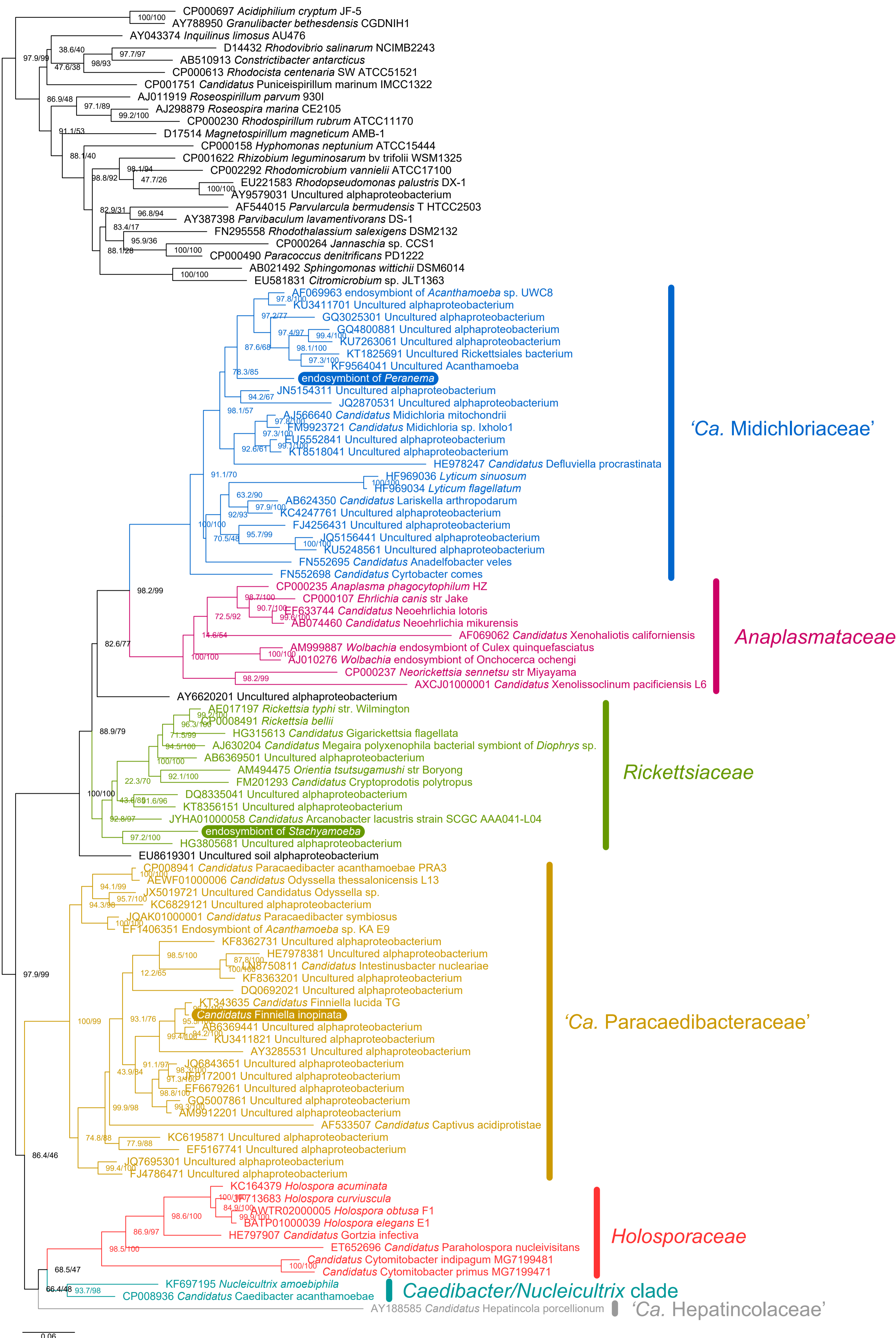

Supplement: Supplementary file 1. — (1) ‘Candidatus Finniella inopinata’ endosymbiont of Viridiraptor invadens strain Virl02, (2) an alphaproteobacterium associated with Peranema trichophorum strain CCAP 1260/1B, and (3) an alphaproteobacterium associated with Stachyamoeba lipophora strain ATCC 50324. Branch support values are SH-aLRT and UFBoot. [file elife-42535-supp1.pdf]
