# Supplementary material for: An updated phylogeny of the Alphaproteobacteria reveals that the parasitic Rickettsiales and Holosporales have independent origins
Source: eLife. 2019 Feb 25;8:e42535. doi: 10.7554/eLife.42535 (PMC6447387; doi:10.7554/eLife.42535)
Supplement: Supplementary file 2. — (A) Ultrafast bootstrap (UFBoot) variation for several clades discussed in this study as compositionally biased sites, according to ɀ, are progressively removed in steps of 10%. (B) Ultrafast bootstrap (UFBoot) variation for several clades discussed in this study as the fastest sites are progressively removed in steps of 10%. (C) GenBank assembly accession numbers for the 120 alphaproteobacterial and outgroup genomes used in this study. (D) A list of the least compositionally heterogeneous genes out of the 200 single-copy and vertically inherited genes used in this study. (E) Model fit of amino acid replacement matrices as components of simple models that do not account for compositional heterogeneity across sites. Models are ordered from lowest to highest BIC. -LnL: log-likelihood; df: degrees of freedom or number of free parameters; AIC: Akaike information criterion; AICc: corrected Akaike information criterion; BIC: Bayesian information criterion. (F) Model fit of amino acid replacement matrices as components of complex models that account for compositional heterogeneity across sites. Models are ordered from lowest to highest BIC. -LnL: log-likelihood; df: degrees of freedom or number of free parameters; AIC: Akaike information criterion; AICc: corrected Akaike information criterion; BIC: Bayesian information criterion. (G) Model fit of LG + ES60+F for which the model component that accounts for rate heterogeneity across sites varies. Models are ordered from lowest to highest BIC. -LnL: log-likelihood; df: degrees of freedom or number of free parameters; AIC: Akaike information criterion; AICc: corrected Akaike information criterion; BIC: Bayesian information criterion. (H) Several summary statistics for the PhyloBayes MCMC chains run for each analysis under the CAT-Poisson+Γ4. [file elife-42535-supp2.docx]

**Supplementary file 1A.** Ultrafast bootstrap (UFBoot) variation for several clades discussed in this study as compositionally-biased sites, according to ɀ, are progressively removed in steps of 10%.

**
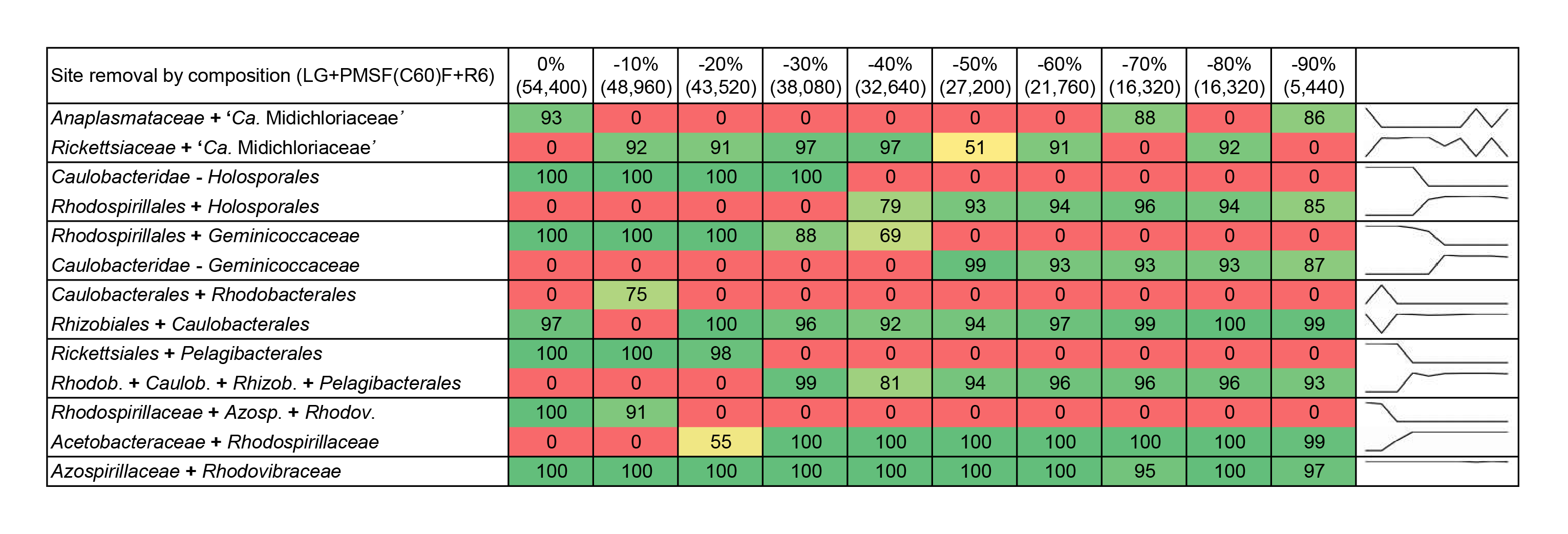
**

**Supplementary file 1B.** Ultrafast bootstrap (UFBoot) variation for several clades discussed in this study as the fastest sites are progressively removed in steps of 10%.

**
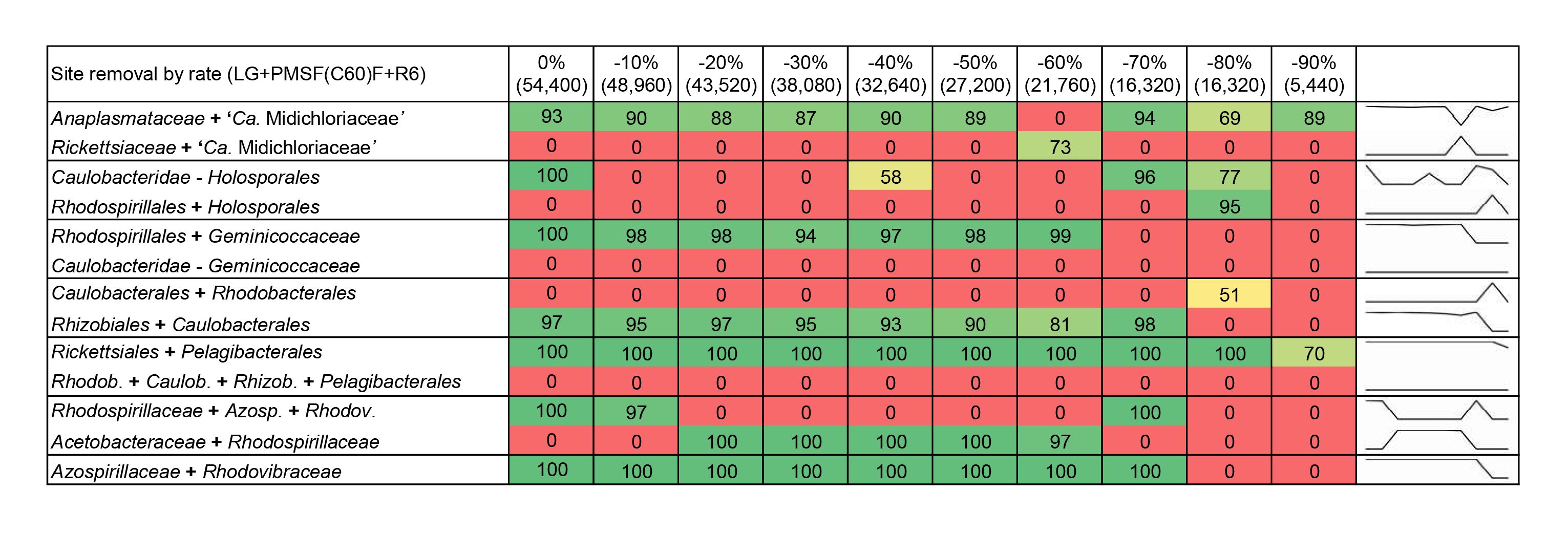
**

**Supplementary file 1C.** GenBank assembly accession numbers for the 120 alphaproteobacterial and outgroup genomes used in this study.

| **Taxon** | **GenBank assembly accession number** |
| --- | --- |
| *Acidiphilium angustum* ATCC 35903 | GCF_000701585.1 |
| *Acidisphaera rubrifaciens* HS-AP3 | GCF_000964365.1 |
| *Ahrensia* sp. R2A130 | GCF_000179775.1 |
| alphaproteobacterium AAP38 | GCF_001296005.1 |
| alphaproteobacterium AAP81b | GCF_001295935.1 |
| alphaproteobacterium BAL199 | GCF_000171835.1 |
| alphaproteobacterium HIMB114 | GCF_000163555.2 |
| alphaproteobacterium HIMB59 | GCF_000299115.1 |
| alphaproteobacterium IMCC14465 | GCF_000293845.2 |
| alphaproteobacterium L41A | GCF_000335735.1 |
| alphaproteobacterium LLX12A | GCF_000335755.1 |
| alphaproteobacterium Mf 1.05b.01 | GCF_000509225.1 |
| alphaproteobacterium Q-1 | GCF_000710935.1 |
| alphaproteobacterium RS24 | GCF_000469155.1 |
| alphaproteobacterium SCGC AAA280-P20 | GCA_000371845.1 |
| alphaproteobacterium SCGC AAA288-N07 | GCA_000513055.1 |
| alphaproteobacterium sp. HIMB5 | GCA_000299095.1 |
| *Alteromonas lipolytica* | GCF_001758465.1 |
| *Anaplasma phagocytophilum* HZ | GCF_000013125.1 |
| *Asticcacaulis excentricus* CB 48 | GCF_000175215.2 |
| *Bartonella quintana* RM-11 | GCA_000294715.1 |
| *Belnapia moabensis* DSM 16746 | GCA_000745835.1 |
| *Brevundimonas subvibrioides* ATCC 15264 | GCA_000144605.1 |
| *Burkholderia thailandensis* E264 | GCA_000012365.1 |
| *Caedibacter* sp. 37-49 | GCA_001898725.1 |
| *Caedibacter* sp. 38-128 | GCA_001898705.1 |
| *Caedibacter varicaedens* | GCA_001192655.1 |
| *Caenispirillum salinarum* AK4 | GCA_000315795.1 |
| *Candidatus* Arcanobacter lacustris | GCA_000970895.1 |
| *Candidatus* Caedibacter acanthamoebae | GCA_000743035.1 |
| *Candidatus* Finniella lucida | This study |
| *Candidatus* Hepatobacter penaei | GCA_000742475.1 |
| *Candidatus* Jidaibacter acanthamoeba | GCA_000815465.1 |
| *Candidatus* Midichloria mitochondrii IricVA | GCA_000219355.1 |
| *Candidatus* Nucleicultrix amoebiphila FS5 | GCA_002117145.1 |
| *Candidatus* Odyssella thessalonicensis L13 | GCA_000190415.2 |
| *Candidatus* Paracaedibacter acanthamoebae isolate PRA3 | GCA_000742835.1 |
| *Candidatus* Paracaedibacter symbiosus | GCA_000757605.1 |
| *Candidatus* Pelagibacter IMCC9063 | GCA_000195085.1 |
| *Candidatus* Pelagibacter ubique HTCC1002 | GCA_000153525.1 |
| *Candidatus* Pelagibacter ubique HTCC8051 | GCA_000472605.1 |
| *Candidatus* Puniceispirillum marinum IMCC1322 | GCA_000024465.1 |
| *Chelativorans* sp. J32 | GCA_000518985.1 |
| *Citromicrobium* sp. JLT1363 | GCA_000186705.2 |
| *Commensalibacter intestini* A911 | GCA_000231445.2 |
| *Congregibacter litoralis* KT71 | GCA_000153125.2 |
| *Ehrlichia canis* Jake | GCA_000012565.1 |
| *Elioraea tepidiphila* DSM 17972 | GCA_000378465.1 |
| endosymbiont of *Peranema* | This study |
| endosymbiont of *Stachyamoeba* | This study |
| *Enterobacter soli* ATCC BAA-2102 | GCA_001654845.1 |
| *Geminicoccus roseus* DSM 18922 | GCA_000427665.1 |
| *Gluconacetobacter diazotrophicus* PA1 5 | GCA_000021325.1 |
| *Gluconobacter oxydans* H24 | GCA_000311765.1 |
| *Granulibacter bethesdensis* CGDNIH1 | GCA_000014285.2 |
| *Hirschia baltica* ATCC 49814 | GCA_000023785.1 |
| *Holospora obtusa* F1 | GCA_000469665.2 |
| *Holospora undulata* HU1 | GCA_000388175.3 |
| *Hyphomicrobium denitrificans* ATCC 51888 | GCA_000143145.1 |
| *Hyphomonas neptunium* ATCC 15444 | GCA_000013025.1 |
| *Inquilinus limosus* DSM 16000 | GCA_000423185.1 |
| *Jannaschia* sp. EhC01 | GCA_001650845.1 |
| *Ketogulonicigenium vulgare* WSH-001 | GCA_000223375.1 |
| *Kiloniella laminariae* DSM 19542 | GCA_000374005.1 |
| *Kordiimonas gwangyangensis* DSM 19435 - JCM 12864 | GCA_000375545.1 |
| *Magnetococcus marinus* MC-1 | GCA_000014865.1 |
| *Magnetofaba australis* IT-1 | GCA_002109495.1 |
| *Magnetospirillum magneticum* AMB-1 | GCA_000009985.1 |
| *Maricaulis maris* MCS10 | GCA_000014745.1 |
| *Meganema perideroedes* DSM 15528 | GCA_000374145.1 |
| *Methylobacterium extorquens* AM1 | GCA_000022685.1 |
| *Methylocella silvestris* BL2 | GCA_000021745.1 |
| *Methylocystis* sp. SC2 | GCA_000304315.1 |
| *Methylovorus glucosetrophus* SIP3-4 | GCA_000023745.1 |
| *Micavibrio aeruginosavorus* ARL-13 | GCA_000226315.1 |
| *Neorickettsia sennetsu* Miyayama | GCA_000013165.1 |
| *Nitrosospira multiformis* ATCC 25196 | GCA_000196355.1 |
| *Oceanibaculum indicum* P24 | GCA_000299935.1 |
| *Oceanicaulis* sp. HTCC2633 | GCA_000152745.1 |
| *Octadecabacter antarcticus* 307 | GCA_000155675.2 |
| *Orientia tsutsugamushi* Boryong | GCA_000063545.1 |
| *Paracoccus denitrificans* PD1222 | GCA_000203895.1 |
| *Pararhodospirillum photometricum* DSM 122 | GCA_000284415.2 |
| *Parvibaculum lavamentivorans* DS-1 | GCA_000017565.1 |
| *Parvularcula bermudensis* HTCC2503 | GCA_000152825.2 |
| *Pelagibacter* sp. HIMB058 | GCF_000012345.1 |
| *Pelagibacterium halotolerans* B2 | GCA_000230555.1 |
| *Phaeospirillum fulvum* MGU-K5 | GCA_000442515.1 |
| *Phenylobacterium zucineum* HLK1 | GCA_000017265.1 |
| *Polymorphum gilvum* SL003B-26A1 | GCA_000192745.1 |
| *Pseudovibrio* sp. FO-BEG1 | GCA_000236645.1 |
| *Ralstonia solanacearum* GMI1000 | GCA_000009125.1 |
| *Rhizobium leguminosarum* bv. trifolii WSM1689 | GCA_000517605.1 |
| *Rhodobacter sphaeroides* 2.4.1 | GCA_000012905.2 |
| *Rhodocista* sp. MIMtkB3 | GCA_001939945.1 |
| *Rhodomicrobium vannielii* ATCC 17100 | GCA_000166055.1 |
| *Rhodopseudomonas palustris* TIE-1 | GCA_000020445.1 |
| *Rhodospirillum centenum* SW | GCA_000016185.1 |
| *Rhodospirillum rubrum* ATCC 11170 | GCA_000013085.1 |
| *Rhodovibrio salinarum* DSM 9154 | GCA_000515255.1 |
| *Rickettsia typhi* Wilmington | GCA_000008045.1 |
| *Roseibium* sp. TrichSKD4 | GCA_000148725.1 |
| *Roseomonas cervicalis* ATCC 49957 | GCA_000164635.1 |
| *Roseospirillum parvum* strain 930I | GCA_900100455.1 |
| *Rubellimicrobium thermophilum* DSM 16684 | GCA_000442315.1 |
| *Rubritepida flocculans* DSM 14296 | GCA_000425365.1 |
| *Ruegeria* sp. ANG-R | GCA_000813985.1 |
| *Sagittula stellata* E-37 | GCA_000169415.1 |
| *Sneathiella glossodoripedis* JCM 23214 | GCA_000616095.1 |
| *Sphingomonas wittichii* | GCA_000016765.1 |
| *Spongiibacter tropicus* DSM 19543 | GCA_000420325.1 |
| *Terasakiella pusilla* DSM 6293 | GCA_000688235.1 |
| *Thalassobaculum salexigens* DSM 19539 | GCA_000423805.1 |
| *Thalassospira profundimaris* WP0211 | GCA_000300275.1 |
| *Thermopetrobacter* sp. TC1 | GCA_000746275.1 |
| *Tistrella mobilis* KA081020-065 | GCA_000264455.2 |
| *Wolbachia* endosymbiont of *Culex quinquefasciatus* Pel | GCA_000073005.1 |
| *Wolbachia* endosymbiont of *Onchocerca ochengi* | GCA_000306885.1 |
| *Xanthobacter autotrophicus* Py2 | GCA_000017645.1 |
| *Zymomonas mobilis* sub mobilis ATCC 10988 | GCA_000175255.2 |

**Supplementary file 1D.** A list of the least compositionally heterogeneous genes out of the 200 single-copy and vertically-inherited genes used in this study.

| **#** | **p-value** | **chi-square** | **Marker** | **Annotation** | **Length** |
| --- | --- | --- | --- | --- | --- |
| 1 | 1.0000 | 1.0000 | Alpha.18 | rpsL 30S ribosomal protein S12 | 123 |
| 2 | 1.0000 | 1.0000 | Alpha.66 | recA recombinase A | 332 |
| 3 | 0.9700 | 1.0000 | Alpha.227 | rpsQ 30S ribosomal protein S17 | 76 |
| 4 | 0.9400 | 1.0000 | Alpha.74 | rplT ribosomal protein L20 | 118 |
| 5 | 0.8600 | 1.0000 | Alpha.34 | rplN 50S ribosomal protein L14 | 122 |
| 6 | 0.8600 | 1.0000 | Alpha.60 | rpsI 30S ribosomal protein S9 | 132 |
| 7 | 0.7900 | 1.0000 | Alpha.77 | rpsS 30S ribosomal protein S19 | 92 |
| 8 | 0.7500 | 1.0000 | Alpha.118 | hypothetical protein | 165 |
| 9 | 0.7400 | 1.0000 | Alpha.69 | 30S ribosomal protein S14 | 101 |
| 10 | 0.7400 | 1.0000 | Alpha.107 | 30S ribosomal protein S11 | 122 |
| 11 | 0.7300 | 1.0000 | Alpha.256 | rpsT 30S ribosomal protein S20 | 87 |
| 12 | 0.6800 | 1.0000 | Alpha.136 | 30S ribosomal protein S10 | 101 |
| 13 | 0.6700 | 1.0000 | Alpha.31 | rplK 50S ribosomal protein L11 | 142 |
| 14 | 0.6700 | 1.0000 | Alpha.36 | rpsD 30S ribosomal protein S4 | 205 |
| 15 | 0.6100 | 1.0000 | Alpha.128 | preprotein translocase subunit YajC | 88 |
| 16 | 0.5500 | 1.0000 | Alpha.65 | rpsM 30S ribosomal protein S13 | 122 |
| 17 | 0.5400 | 1.0000 | Alpha.123 | nuoK2 NuoK2 NADH quinone oxidoreductase subunit 11 (chain K) | 102 |
| 18 | 0.4700 | 1.0000 | Alpha.42 | NAD(P)H-quinone oxidoreductase subunit 3 | 120 |
| 19 | 0.4500 | 1.0000 | Alpha.79 | Ribosomal protein L19 | 121 |
| 20 | 0.4400 | 1.0000 | Alpha.70 | rpsH 30S ribosomal protein S8 | 132 |
| 21 | 0.4200 | 1.0000 | Alpha.68 | rpmA 50S ribosomal protein L27 | 84 |
| 22 | 0.3600 | 1.0000 | Alpha.99 | rplM 50S ribosomal protein L13 | 154 |
| 23 | 0.3600 | 1.0000 | Alpha.172 | ATP-dependent HslUV protease peptidase subunit HslV | 176 |
| 24 | 0.3300 | 1.0000 | Alpha.9 | glutaredoxin-like protein grla | 106 |
| 25 | 0.3200 | 1.0000 | Alpha.37 | rpsC 30S ribosomal protein S3 | 213 |
| 26 | 0.2900 | 1.0000 | Alpha.233 | transcriptional regulator | 163 |
| 27 | 0.2800 | 1.0000 | Alpha.12 | 30S ribosomal protein S7 | 156 |
| 28 | 0.2700 | 1.0000 | Alpha.147 | rpsR 30S ribosomal protein S18 | 73 |
| 29 | 0.2600 | 1.0000 | Alpha.67 | DNA-directed RNA polymerase subunit alpha | 331 |
| 30 | 0.2400 | 1.0000 | Alpha.210 | 30S ribosomal protein S6 | 105 |
| 31 | 0.2100 | 1.0000 | Alpha.117 | rpsP 30S ribosomal protein S16 | 103 |
| 32 | 0.2100 | 1.0000 | Alpha.235 | ppa inorganic pyrophosphatase | 174 |
| 33 | 0.1600 | 1.0000 | Alpha.56 | hypothetical protein | 213 |
| 34 | 0.1200 | 1.0000 | Alpha.93 | rplQ 50S ribosomal protein L17 | 124 |
| 35 | 0.1000 | 1.0000 | Alpha.170 | nucleoside diphosphate kinase | 140 |
| 36 | 0.1000 | 1.0000 | Alpha.215 | iojap protein family | 103 |
| 37 | 0.0900 | 1.0000 | Alpha.39 | rplE 50S ribosomal protein L5 | 179 |
| 38 | 0.0900 | 1.0000 | Alpha.73 | rplV 50S ribosomal protein L22 | 116 |
| 39 | 0.0600 | 1.0000 | Alpha.41 | infC translation initiation factor IF-3 | 165 |
| 40 | 0.0600 | 1.0000 | Alpha.149 | 30S ribosomal protein S15 | 89 |

**Supplementary file 1E.** Model fit of amino acid replacement matrices as components of simple models that do not account for compositional heterogeneity across sites. Models are ordered from lowest to highest BIC. -LnL: log-likelihood; df: degrees of freedom or number of free parameters; AIC: Akaike information criterion; AICc: corrected Akaike information criterion; BIC: Bayesian information criterion.

| **Model** | **-LnL** | **df** | **AIC** | **AICc** | **BIC** |
| --- | --- | --- | --- | --- | --- |
| Poisson+F+G4 | 7659896.621 | 257 | 15320307.242 | 15320309.691 | 15322595.601 |
| Dayhoff+F+G4 | 7091431.338 | 257 | 14183376.676 | 14183379.125 | 14185665.035 |
| JTT+F+G4 | 7078685.705 | 257 | 14157885.411 | 14157887.860 | 14160173.769 |
| BLOSUM62+F+G4 | 7054099.636 | 257 | 14108713.272 | 14108715.721 | 14111001.631 |
| WAG+F+G4 | 7006674.211 | 257 | 14013862.421 | 14013864.870 | 14016150.780 |
| LG+F+G4 | 6975286.517 | 257 | 13951087.035 | 13951089.484 | 13953375.394 |

**Supplementary file 1F.** Model fit of amino acid replacement matrices as components of complex models that account for compositional heterogeneity across sites. Models are ordered from lowest to highest BIC. -LnL: log-likelihood; df: degrees of freedom or number of free parameters; AIC: Akaike information criterion; AICc: corrected Akaike information criterion; BIC: Bayesian information criterion.

| **Model** | **-LnL** | **df** | **AIC** | **AICc** | **BIC** |
| --- | --- | --- | --- | --- | --- |
| Poisson+ES60+F+R6 | 6879393.128 | 326 | 13759438.256 | 13759442.199 | 13762340.999 |
| Dayhoff+ES60+F+R6 | 6759599.477 | 326 | 13519850.954 | 13519854.897 | 13522753.697 |
| JTT+ES60+F+R6 | 6744047.628 | 326 | 13488747.256 | 13488751.199 | 13491649.999 |
| BLOSUM62+ES60+F+R6 | 6732833.254 | 326 | 13466318.509 | 13466322.452 | 13469221.252 |
| WAG+ES60+F+R6 | 6725632.546 | 326 | 13451917.093 | 13451921.036 | 13454819.836 |
| LG+ES60+F+R6 | 6723504.529 | 326 | 13447661.058 | 13447665.001 | 13450563.801 |

**Supplementary file 1G.** Model fit of LG+ES60+F for which the model component that accounts for rate heterogeneity across sites varies. Models are ordered from lowest to highest BIC. -LnL: log-likelihood; df: degrees of freedom or number of free parameters; AIC: Akaike information criterion; AICc: corrected Akaike information criterion; BIC: Bayesian information criterion.

| **Model** | **-LnL** | **df** | **AIC** | **AICc** | **BIC** |
| --- | --- | --- | --- | --- | --- |
| LG+ES60+F+G | 6742494.385 | 317 | 13485622.770 | 13485626.498 | 13488445.376 |
| LG+ES60+F+R4 | 6739954.613 | 322 | 13480553.226 | 13480557.073 | 13483420.352 |
| LG+ES60+F+R5 | 6730038.899 | 324 | 13460725.798 | 13460729.693 | 13463610.733 |
| LG+ES60+F+R6 | 6723504.529 | 326 | 13447661.058 | 13447665.001 | 13450563.801 |

**Supplementary file 1H.** Several summary statistics for the PhyloBayes MCMC chains run for each analysis under the CAT-Poisson+Γ4.

| **Figure/PhyloBayes analysis** | **Parameter** | **Effective sample size** | **Discrepancy** |
| --- | --- | --- | --- |
| **Fig. S4A** Chain 1: 9165 cycles Chain 2: 9160 cycles maxdiff: 1 meandiff: 0.0173201 | loglik | 19 | 0.1234140 |
|  | length | 130 | 3.2251700 |
|  | alpha | 117 | 0.2423630 |
|  | Nmode | 49 | 0.1955280 |
|  | statent | 38 | 0.0286658 |
|  | statalpha | 21 | 0.0002796 |
| **Fig. S4B**  Chain 1: 43770 cycles Chain 2: 34846 cycles maxdiff: 0.438046 meandiff: 0.0026012 | loglik | 133 | 0.0273831 |
|  | length | 866 | 0.0332865 |
|  | alpha | 932 | 0.0736650 |
|  | Nmode | 267 | 0.0311482 |
|  | statent | 199 | 0.0898815 |
|  | statalpha | 506 | 0.0661686 |
| **Fig. S11A**  Chain 1: 15651 cycles Chain 2: 15566 cycles maxdiff: 0.0272492 meandiff: 0.00014417 | loglik | 107 | 0.0384224 |
|  | length | 272 | 0.0007326 |
|  | alpha | 615 | 0.0854009 |
|  | Nmode | 103 | 0.0315492 |
|  | statent | 223 | 0.1356220 |
|  | statalpha | 452 | 0.0614499 |
| **Fig. S11B**  Chain 1: 28047 cycles Chain 2: 26102 cycles maxdiff: 0.0804344 meandiff: 0.00285125 | loglik | 158 | 0.0584390 |
|  | length | 132 | 0.1859400 |
|  | alpha | 172 | 0.0255812 |
|  | Nmode | 2972 | 0.0242992 |
|  | statent | 122 | 0.0597558 |
|  | statalpha | 3712 | 0.0133815 |
| **Fig. S13**  Chain 1: 20121 cycles Chain 2: 20874 cycles maxdiff: 0.100318 meandiff: 0.00167925 | loglik | 10767 | 0.0546108 |
|  | length | 4357 | 0.0613948 |
|  | alpha | 891 | 0.0160582 |
|  | Nmode | 1913 | 0.0870560 |
|  | statent | 3092 | 0.0017995 |
|  | statalpha | 2457 | 0.0203763 |
